# Differences in Hamstring Muscle‐Tendon Unit Geometry and Function Between Elite Sprint and Jump Athletes and Recreationally Active Controls

**DOI:** 10.1111/sms.70151

**Published:** 2025-10-24

**Authors:** Stephanie L. Lazarczuk, Andrea H. Hams, Phillip M. Bellinger, Ryan G. Timmins, Eline Lievens, Ben Kennedy, David Opar, Rod S. Barrett, Matthew N. Bourne

**Affiliations:** ^1^ Department of Sport and Health Southampton Solent University Southampton UK; ^2^ School of Allied Health, Sport and Social Work Griffith University Gold Coast Queensland Australia; ^3^ Australian Centre for Precision Health and Technology Griffith University Gold Coast Queensland Australia; ^4^ Griffith Sports Science Griffith University Gold Coast Queensland Australia; ^5^ School of Behavioural and Health Sciences Australian Catholic University Brisbane Queensland Australia; ^6^ Sports Performance, Recovery, Injury and New Technologies (SPRINT) Research Centre Australian Catholic University Melbourne Victoria Australia; ^7^ Department of Movement and Sports Sciences Ghent University Ghent Belgium; ^8^ Mermaid Beach Radiology Gold Coast Queensland Australia; ^9^ School of Behavioural and Health Sciences Australian Catholic University Melbourne Victoria Australia

**Keywords:** aponeurosis, biceps femoris, semimembranosus, semitendinosus, spectroscopy

## Abstract

The hamstrings are critical for athletic performance; however, no study has examined differences in hamstring muscle‐tendon geometry (cross‐sectional area/volume) and muscle typology (proportion of Type I/II fibers) between elite sprinters/jumpers and recreationally active individuals. This study aimed to compare hamstring geometry and typology between these groups and examine how these characteristics relate to sprint and strength performance. Elite sprint and jump athletes (*n* = 15, 3 female, 21.7 ± 2.2 y, 180.6 ± 9.9 cm, 72.2 ± 9.6 kg) and recreationally active individuals (*n* = 15, 4 female, 25.7 ± 3.0 y, 176.0 ± 9.5 cm, 76.3 ± 17.6 kg) completed sprint and eccentric knee flexor strength testing. Magnetic resonance imaging and spectroscopy were used to assess hamstring muscle‐tendon geometry and typology, respectively. Compared to recreationally active individuals, elite athletes had larger hamstring muscles (all muscles, mean difference: 59.75–150.45 cm^3^, *p* < 0.009), biceps femoris long head (BFlh) proximal aponeuroses (1.09 cm^3^, *p* < 0.001), BF short head distal aponeuroses (1.24 cm^3^, *p* = 0.002), semimembranosus proximal free tendons (0.75 cm^3^, *p* = 0.024) and aponeuroses (2.29 cm^3^, *p* < 0.001), semitendinosus distal free tendons (0.49 cm^3^, *p* = 0.01) and BFlh proximal aponeurosis interface areas (10.43 cm^2^, *p* < 0.001). Elite athletes also had 1.5 times greater estimated proportion of Type II fibers (*p* < 0.001). Medial hamstring geometry and muscle typology explained the greatest variance in maximal sprint speed (*R* ^2^ = 0.65), while BFlh and semimembranosus muscle volumes with semitendinosus tendon volume explained the greatest variance in eccentric knee flexor strength (*R* ^2^ = 0.59). Elite athletes had larger hamstring muscles, aponeuroses, and free tendons, and a greater estimated proportion of Type II fibers than recreationally active individuals. These structural and compositional differences likely contribute to their superior sprint and strength performance.

## 1 Introduction

The hamstrings play a crucial role in high‐speed running by generating large hip extensor moments during the propulsive stance phase and rapidly decelerating the shank during terminal swing [1, 2, 3]. These actions have implications for injury susceptibility given that hamstring strain injuries (HSIs) remain the number one injury in running‐based sports [4]. Hamstring function is significantly influenced by muscle‐tendon unit geometry [5, 6] and the proportion of Type I and II fibers (i.e., muscle typology), which can differentiate athletic performance ability [7, 8] and is related to strength qualities [9]. While differences in hamstring muscle geometry between sprint athletes and other populations have been described [5, 6, 10], no study has examined the corresponding free tendons and aponeuroses or explored how these factors relate to performance (i.e., sprint and strength abilities).

Sprinters typically have larger hamstring muscles than non‐elite athletes or non‐athletes [5, 6, 10]. Handsfield et al. reported that male sprinters' semitendinosus muscles were 54% larger, biceps femoris long head (BFlh) and short head (BFsh) 26% larger, and the semimembranosus was 20% larger than recreationally active individuals [5]. Additionally, Kawama et al. demonstrated that improvements in sprint time over a one‐year period were positively correlated with increased semitendinosus muscle volume relative to mass [11]. Prior work has established relationships between hamstring muscle volume and maximal strength qualities (*r* = 0.62–0.76, *p* < 0.01) which can underpin athletic performance [9]. However, muscle volume alone may not capture non‐uniform hypertrophic adaptations. We have previously shown that 10 weeks of training with the Nordic hamstring or hip extension exercise resulted in regional increases in hamstring muscle cross‐sectional area (CSA) [12] but this nuance may not be clear based on volume alone. Similarly, Kawama et al. (2021) observed that sub‐elite sprinters had larger proximal and middle portions of semitendinosus and a larger middle portion of BFsh than university‐level rugby players [10]. Additionally, the distal portions of BFlh, semitendinosus and semimembranosus were larger in sprinters than non‐athletes [10]. Such non‐uniform adaptations may influence running mechanics, as greater proximal thigh muscle mass is thought to be less costly on the thigh's moment of inertia compared to distal hypertrophy [5, 13]. Understanding how long‐term mechanical loading affects regional muscle development may provide insight into how sprint athletes generate high sprint velocities.

Short‐term (i.e., 10‐weeks) resistance training using the Nordic or hip extension exercises promotes significant hamstring muscle hypertrophy and increased eccentric knee flexor strength but did not stimulate changes in free tendon or aponeurosis volume [12]. Larger and presumably stiffer healthy lower limb tendons [14] store more energy at any given strain and experience less strain at a given load. Stiffer lower limb tendons are associated with higher rates of force development [15], improved running economy [16] and faster sprint times [17]. Additionally, the geometry and relative sizes of the BFlh muscle and aponeurosis can influence muscle fiber strain at the commonly injured proximal musculotendinous junction (MTJ) [18, 19]. A wider BFlh muscle and narrower aponeurosis was estimated to increase peak muscle fiber strain near to the proximal MTJ [18, 19]. Although experimental evidence is lacking, a relatively larger interface area between muscle and aponeurosis could provide a greater area for muscular attachment and force distribution, potentially reducing the stress and strain experienced by muscle fibers and/or tendons. Lower limb tendons are sensitive to mechanical loading [20], and a cross‐sectional study of trained weightlifters and untrained students demonstrated that both the vastus lateralis muscle and aponeurosis were larger in the trained cohort [21], suggesting that habitual training may lead to larger aponeuroses alongside hypertrophied muscles. Consequently, it is likely that habitual loading over many months or years is required to induce significant tendon‐based geometric alterations [22, 23] and studying athletes exposed to long‐term hamstring loading could offer valuable insights into the adaptive potential of tendons and aponeuroses.

Further to muscle‐tendon geometry, muscle typology has been linked to injury risk and performance. Faster athletes tend to possess a greater proportion of Type II (i.e., fast) than Type I (i.e., slow) fibers [8]. Our previous work showed that gastrocnemius muscle typology—estimated non‐invasively via proton magnetic resonance spectroscopy—is a strong determinant of force and velocity in sprinting and jumping [7]. Using this technique, Lievens and colleagues reported that footballers with a greater proportion of Type II fibers were 5.3‐fold more likely to sustain a HSI than those with predominantly Type I fibers [24]. Additionally, confocal microscopy revealed that the footprint (i.e., direct insertion area) of isolated semitendinosus fibers was 22% larger in Type I than Type II fibers [25]. This raises the possibility that Type I fibers could be more resistant to strain injury than Type II fibers which support explosive, high‐force activities. However, no study has determined whether BFlh muscle typology differs between elite athletes and untrained individuals or if it is associated with sprint performance. Additionally, despite evidence suggesting that the size of the aponeurosis [18] and individual fiber types [25] may influence risk of injury, the relationship between BFlh muscle typology and the muscle‐aponeurosis interface area has yet to be explored.

The primary aims of this study were to assess differences in (1) the volumes and regional CSAs of the hamstring muscles, free tendons, and aponeuroses, and (2) the BFlh interface area and BFlh muscle volume‐to‐aponeurosis interface area ratio between elite sprinters/jumpers and recreationally active controls. We hypothesized that long‐term trained athletes would have larger hamstring muscle, free tendon, and aponeurosis volumes, accompanied by larger interface areas. Additionally, we hypothesized that there would be muscle‐specific differences in the regional CSA of hamstring muscles. The secondary aim of the study was to explore the association between hamstring muscle‐tendon unit geometry and BFlh carnosine content on performance during sprint and strength testing.

## 2 Methods

### 2.1 Study Design

This cross‐sectional study compared long‐term trained, elite athletes to a recreationally active group. Participants completed two data collection sessions separated by approximately 5–10 days. Sprint and eccentric knee flexor strength data were acquired during the first session, and magnetic resonance imaging (MRI) scans of both lower limbs were obtained at a radiology clinic in the second. Ethical approval was granted by the Griffith University Human Research Ethics Committee (reference number: GU 2020/508).

### 2.2 Participants

Fifteen elite sprint or jump athletes (3 female, 21.7 ± 2.2 years, 180.6 ± 9.9 cm, 72.2 ± 9.6 kg) and 15 recreationally active individuals (4 female, 25.7 ± 3.0 years, 176.0 ± 9.5 cm, 76.3 ± 17.6 kg) participated. Inclusion criteria for all participants were: any gender, between 18 and 40 years, free from musculoskeletal injury for ≥ 3 months, no history of Grade 2 HSI or above (self‐reported following assessment by a medical practitioner or no longer than 7 days of competition/training activity lost to hamstring‐related pain), no history of traumatic knee injury (e.g., anterior cruciate ligament rupture), and no contraindications to MRI. Participants were excluded if they consumed carnosine or beta‐alanine supplements, were vegetarian or vegan. Participants in the elite group were required to be competing at state level or higher in a sprint or horizontal jumps event and consistently training on track and gym environments (i.e., weekly ≥ 5 sessions combined). Training histories for participants are reported in Supporting Information S1. Recreationally active controls were required to have completed no structured lower limb resistance training for ≥ 1 year, and to not be regularly training or competing in field or court sports, or any other sport involving sprinting.

### 2.3 Performance Testing

Participants completed a 15 min, self‐directed warm up, culminating in progressive increases in running speed over three 60 m efforts (e.g., 50%, 75%, 90% of perceived maximal effort). Participants completed six maximal sprint efforts on an outdoor athletics track, separated by up to 7 min of inter‐effort rest. Maximal sprint velocity was captured using a radar gun (Stalker ATS Pro II). Three efforts focussed on maximum acceleration from a stationary start and maintaining maximal sprint velocity, followed by three efforts with a rolling start with more progressive acceleration to, and maintenance of, maximal sprint velocity from approximately 30 m. Maximum sprint velocity from all trials was used for subsequent analysis.

Eccentric knee flexor force was measured using a NordBord (Vald Performance, Australia). Participants completed three submaximal warm up attempts (e.g., 50%, 75%, 90% of perceived maximal effort), followed by three maximal measured attempts. Participants were provided with 2 min inter‐repetition rest between attempts. Participants were offered coaching points during submaximal attempts and continued encouragement through measured attempts. Participants kneeled on the device and lowered themselves to the ground as slowly as possible, using their arms to push back to the start position. If participants could control the descent through the full range (i.e., with no break‐point or fall), they completed up to three additional repetitions with a weight plate held to their chest (centered at the xiphoid process) in increments of 5 kg until they either could not control the full range or had no further increase in eccentric force versus the previous trial. Peak eccentric knee flexor force was extracted for subsequent analysis.

### 2.4 Magnetic Resonance Imaging and Spectroscopy Acquisition

Participants attended a local radiology clinic within 10 days of performance testing, where images of both thighs were acquired using a 3 Tesla (3 T) MRI scanner (Ingenia Elition X, Philips Healthcare, Table 1). To minimize the effects of muscle damage or anomalous fluid shifts, participants were instructed to avoid lower limb resistance training or sprints in the 48 h preceding acquisition and were seated for approximately 15 min before image acquisition. Participants were positioned feet‐first in the scanner, in supine with their hips in neutral and knees extended. Contiguous, axial T1‐weighted images were acquired for the full lower limb (i.e., iliac crest to toes). Scans were reconstructed using Smart Speed AI reconstruction (Phillips, Netherlands). Muscle carnosine concentration of the right BFlh and medial gastrocnemius was assessed using proton magnetic resonance spectroscopy using previously established methods (Table 1) [26].

**TABLE 1:** Acquisition parameters for magnetic resonance imaging and spectroscopy.

Parameter	Anatomical imaging	Spectroscopy
Coil	Spinal	Knee (gastrocnemius), Spinal (BFlh)
Acquisition technique	Fast field echo (FFE) mDixon multi‐echo	SV Echo
Method	—	sLASER
Spectral Bandwidth	—	2000 Hz
Phase direction	Left–right	—
Compress sense	2	—
Slice oversampling	1.5	—
Slice orientation	Transverse	—
Slice thickness	2 mm	4 mm
Interslice gap	0.2 mm	0.4 mm
Repetition time	5 ms	2000 ms
Echo time	Echo 1 = 1.94, Echo 2 = 3.4	Shortest 44 ms
Water‐fat shift	0.407	—
Flip angle (deg)	10	—
Half scan	Partial Fourier, *Y* = 0.7, *Z* = 0.75	—
Field of view (mm)	260 × 450	—
Acquisition matrix	260 × 450	280 × 126
Voxel size	1 × 1 × 2 mm (reconstructed 0.439 × 0.439)	20 × 20 × 40 mm
Scan duration	1:56 min	5:56 min
Phase cycles	—	16
Samples	1 NSA	2048, 160 NSA
RF Shims	—	Adaptive, using multi‐transmit B1

Abbreviation: NSA, number of spectrum averages.

### 2.5 Performance Data Processing

Sprint data were smoothed using a moving‐window average, and maximal velocity (m/s) was determined for each trial. Strength testing data were processed automatically by the proprietary Vald software to extract peak eccentric knee flexor force (N) for the Nordic exercise (two‐limb average). Peak eccentric force was normalized to body mass (N/kg) to allow between‐group comparisons.

### 2.6 Image Processing

One author (SLL) manually segmented all hamstring muscles and their associated proximal and distal tendons (free tendon and aponeuroses) on axial MRI slices using Materialise Interactive Medical Image Control System software (Mimics, v25.0, Materialise). Reliability for manual segmentation by the author has been previously published (ICC (3, 1) = 0.995, 95% CI = 0.991–0.997) [27]. All structures were segmented from the first slice in which they were visible, through to the last in which they could be visualized. CSA was extracted from each segmented axial slice, for each tissue. Muscle, free tendon, and aponeurosis volumes were calculated as the product of slice thickness and summed axial CSAs. Femurs were segmented bilaterally for inclusion as a model random effect. Interface length (the contact border between the BFlh muscle and aponeurosis) was manually traced by one author (SLL) on each axial slice in which it was visible using the ‘spline’ function [12]. The interface area was calculated as the product of summed interface surface lengths and slice thickness [12].

### 2.7 Spectroscopy Processing

Carnosine content was determined based on the magnitude of the carnosine peak at 8 ppm. For the gastrocnemius, the absolute carnosine concentration was established as previously described [26], with correction factors applied for the T1 and T2 relaxation times of the muscle. For the BFlh, correction factors were not available, therefore carnosine peaks were expressed relative to the water peak as a ratio value. Test–retest reliability and average coefficient of variation between duplicate measures was determined for the BFlh carnosine peak‐to‐water peak values in 7 additional participants (Supporting Information S2). The average coefficient of variation (CV) between repeat measures of the carnosine peak‐to‐water peak value was 11%. The CV for gastrocnemius carnosine content repeat measures has been reported as 4.3% [26].

### 2.8 Statistical Analysis

All statistical analyses were performed in R (v4.3.2, in R Studio) [28]. Participant characteristic data were assessed for normality using the Shapiro–Wilk test, and between‐group comparisons conducted using a *t*‐test. Linear mixed effects models using the ‘lme4’ package [29] were used to assess the effect of group (elite, recreationally active) on all hamstring muscle, free tendon, and aponeurosis volumes, BFlh interface area, BFlh muscle volume‐to‐interface area ratio, and muscle typology. For each outcome measure a linear mixed effects model was fitted using the following formula: value ~ group + mass + gender + age + average femur length + (1|ID). ‘Value’ relates to volume, interface area or ratio, while ID relates to the participant alphanumeric identifier. Group was included in the model as an interacting fixed effect, with mass, gender, age, and average femur length included as fixed effects to account for expected differences in participant anthropometrics. The participant identifier was included as a random effect to enable multiple sample sites to be included, with left and right lower limbs, and both proximal and distal free tendons or aponeuroses. Post hoc analyses of the linear mixed effects models were performed using the ‘emmeans’ package [30]. Contrasts for the main effect of group were performed using pairwise comparisons. Statistical significance for between group differences was set at *p* = 0.05.

Regional differences in muscle CSA were assessed using one‐dimensional statistical parametric mapping (SPM). Muscles were normalized to 0%–100% of their lengths using linear interpolation where the number of data points was based on the median number of slices across participants for each tissue. In Python, the ‘spm1d’ package [31] was used, performing a *t*‐test to compare between groups.

Stepwise forward regression analyses were conducted using the ‘step’ function in R (v4.3.2, in R Studio) [28] to assess the influence of geometric muscle‐tendon unit variables and BFlh carnosine peak‐to‐water peak values on sprint and absolute knee flexor strength performance. Muscle volume for each hamstring was included as a potential variable, alongside the sum of proximal and distal free tendon and aponeurosis volumes for each hamstring (e.g., BFsh tendon volume = distal aponeurosis volume + distal free tendon volume). BFlh carnosine peak‐to‐water peak was included in the regression analysis for both sprint and knee flexor strength performance. Knee flexor strength was also included as a potential variable for sprint performance.

## 3 Results

### 3.1 Participant Characteristics

Elite participants were significantly younger, faster, and stronger than the recreationally active group. There was no difference in height or mass between groups (Table 2).

**TABLE 2:** Participant characteristics and performance outcome measures.

	Elite	Recreationally active
Age (y)**	21.7 ± 2.2	25.7 ± 3.0
Height (cm)	180.6 ± 9.9	176.0 ± 9.5
Mass (kg)	72.2 ± 9.6	76.3 ± 17.6
Male/Female	12/3	11/4
Maximal sprint velocity (m/s)**	10.17 ± 0.79^a^	7.77 ± 0.86^b^
Peak Nordic force (N)*	395.7 ± 126.0	277.1 ± 102.3
Normalized peak Nordic force (N/kg)***	5.42 ± 1.41	3.67 ± 1.18

*Note:* Values are expressed as mean ± standard deviation. The average of peak force for each limb is presented for Nordic force measures. Male/female is expressed as *n*.

^a^ *n* = 2 sprint velocities unavailable.

^b^ *n* = 3 sprint velocities unavailable.

* *p* < 0.01.

** *p* < 0.001.

*** *p* = 0.001.

### 3.2 Hamstring Muscle, Free Tendon, and Aponeurosis Volumes

**FIGURE 1:**
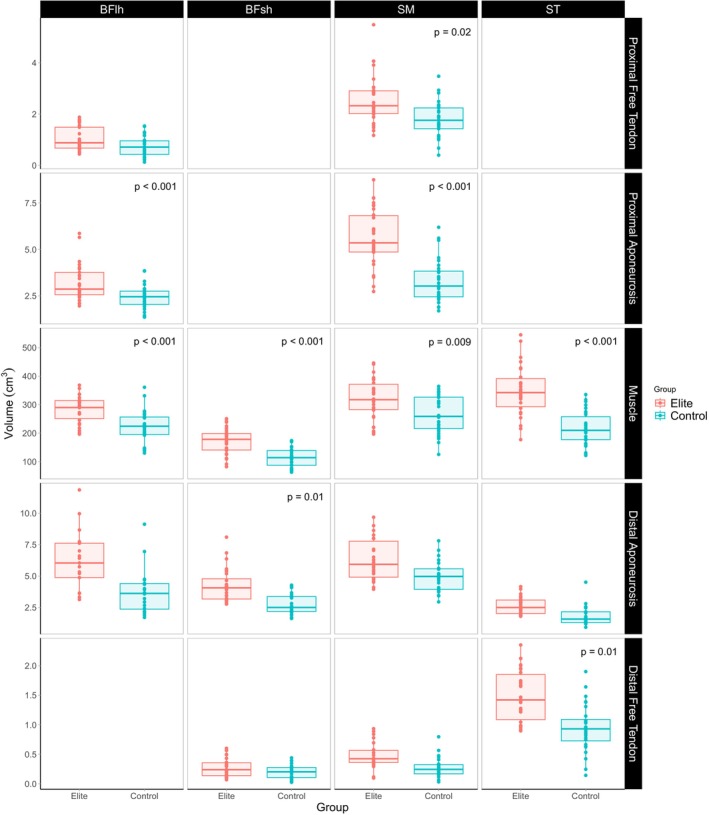
Boxplots showing the volume of the proximal and distal aponeuroses and free tendons and muscle of each hamstring muscle‐tendon unit, for the elite and recreationally active control groups. Dots represent the individual limbs of participants. Error bars represent 95% confidence intervals. BFlh, biceps femoris long head; BFsh, biceps femoris short head; SM, semimembranosus; ST, semitendinosus.

The elite group had significantly larger BFlh muscle (mean difference: 78.46 cm^3^, 95% CI = 51.88–105.05 cm^3^, *p* < 0.001; Figure 1, raw values presented in Supporting Information S5.1) and proximal aponeurosis volumes (mean difference: 1.09 cm^3^, 95% CI = 0.46–1.72 cm^3^, *p* = 0.002) than the recreationally active group. Further, the elite group possessed significantly larger BFsh muscle (mean difference: 62.08 cm^3^, 95% CI = 37.56–86.60 cm^3^, *p* < 0.001) and distal aponeurosis volumes (mean difference: 1.24 cm^3^, 95% CI = 0.27–2.21 cm^3^, *p* = 0.015). The elite group had significantly larger semimembranosus proximal free tendons (mean difference: 0.75 cm^3^, 95% CI = 0.11–1.38 cm^3^, *p* = 0.024), proximal aponeuroses (mean difference: 2.29 cm^3^, 95% CI = 1.30–3.28 cm^3^, *p* < 0.001) and muscle volumes (mean difference: 59.75 cm^3^, 95% CI = 16.68–102.82 cm^3^, *p* = 0.009) than the recreationally active group. Finally, the elite group also possessed significantly larger semitendinosus muscle (mean difference: 150.45 cm^3^, 95% CI = 104.40–196.49 cm^3^, *p* < 0.001) and distal free tendon (mean difference: 0.49 cm^3^, 95% CI = 0.13–0.84 cm^3^, *p* = 0.010) volumes than the recreationally active group.

### 3.3 BFlh Aponeurosis Interface Area, Muscle‐to‐Aponeurosis Interface Ratio and Carnosine Content

**FIGURE 2:**
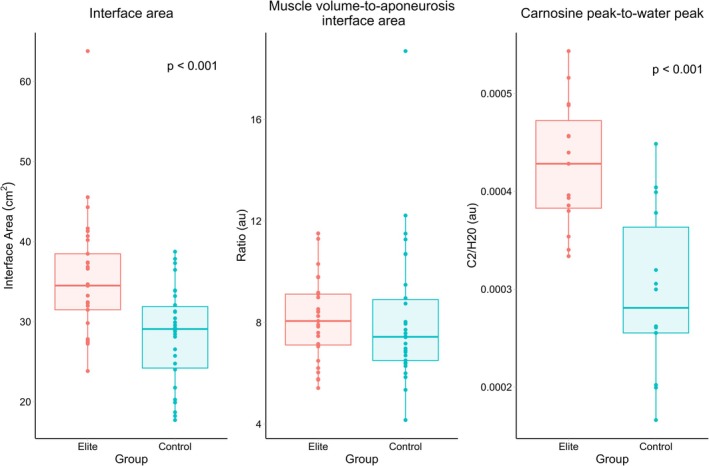
Boxplots showing the biceps femoris long head interface area, muscle volume‐to‐aponeurosis interface area ratio, and the carnosine‐to‐water peak values for the elite and recreationally active control groups. Dots represent the individual limbs of participants. Error bars represent 95% confidence intervals.

The elite group had significantly larger BFlh proximal aponeurosis interface areas than the recreationally active group (mean difference: 10.43 cm^2^, 95% CI = 5.33–15.54 cm^2^, *p* < 0.001; Figure 2). There were no significant differences between groups for the BFlh muscle volume‐to‐aponeurosis interface area ratio (*p* = 0.73).

**FIGURE 3:**
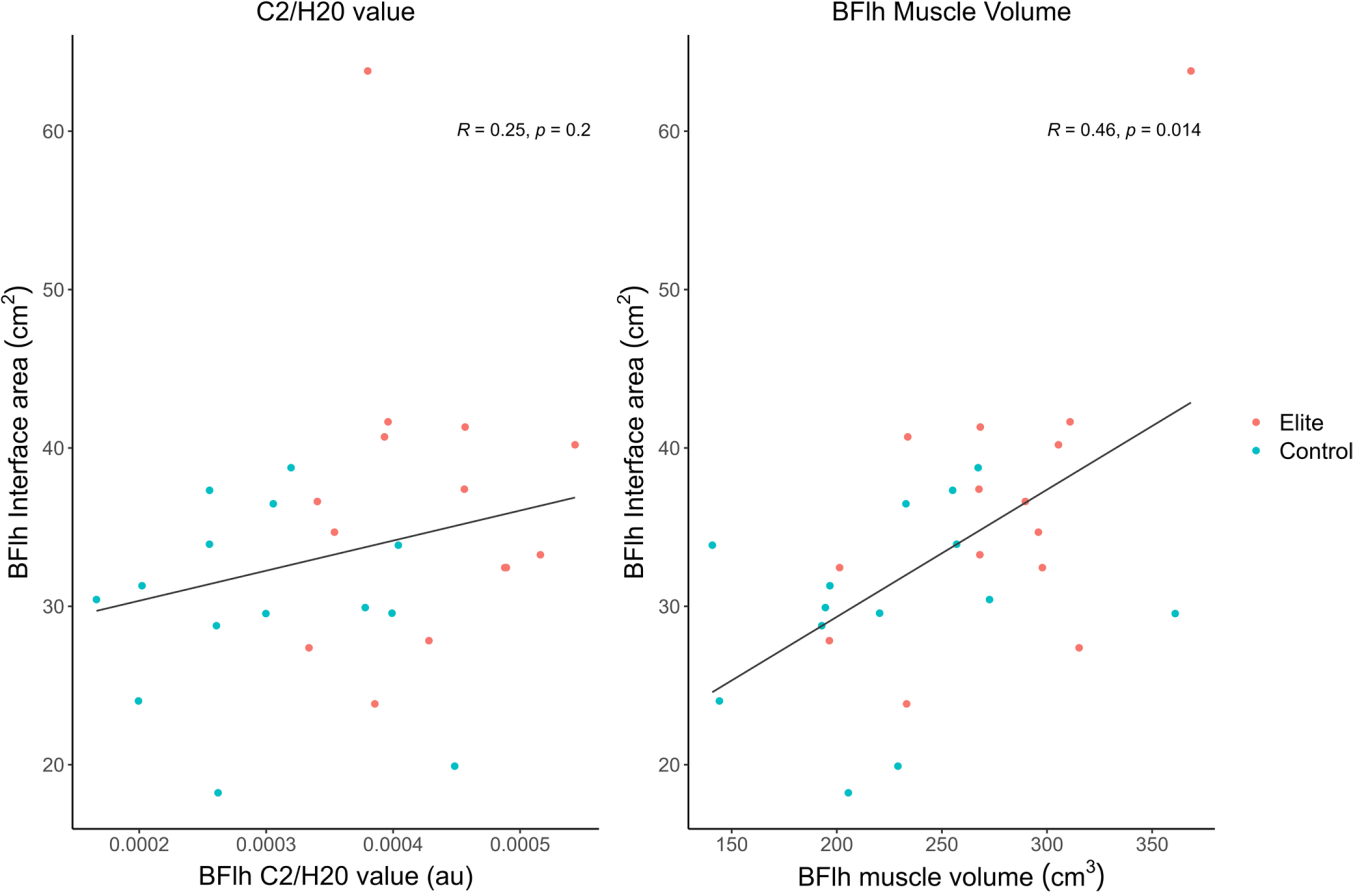
Correlations between biceps femoris long head (BFlh) carnosine peak‐to‐water peak (C2/H20 value) and interface area, and BFlh muscle volume and interface area. Dots represent the right leg of individual participants. Spearman's *ρ* (*R*) and *p* value are also presented.

The elite group had a significantly higher BFlh carnosine peak relative to water peak value (mean difference: 0.00015 au, 95% CI: 0.0001–0.0002 au, *p* < 0.001), being 1.5 times greater than the recreationally active group. The BFlh carnosine peak‐to‐water peak value and the interface area was not significantly correlated (*ρ* = 0.25, *p* = 0.20; Figure 3). There was a significant, positive correlation between the BFlh interface area and muscle volume (*ρ* = 0.46, *p* = 0.01, Figure 3). The elite group also had a significantly higher gastrocnemius carnosine content than the recreationally active group (mean difference: 2.52 mM, 95% CI: 0.87–2.52 mM, *p* = 0.004). Additionally, there was a significant, positive correlation between gastrocnemius carnosine content and BFlh carnosine peak‐to‐water peak (*ρ* = 0.66, *p* = 0.01; Supporting Information S4).

### 3.4 Regional Differences in Muscle CSA

**FIGURE 4:**
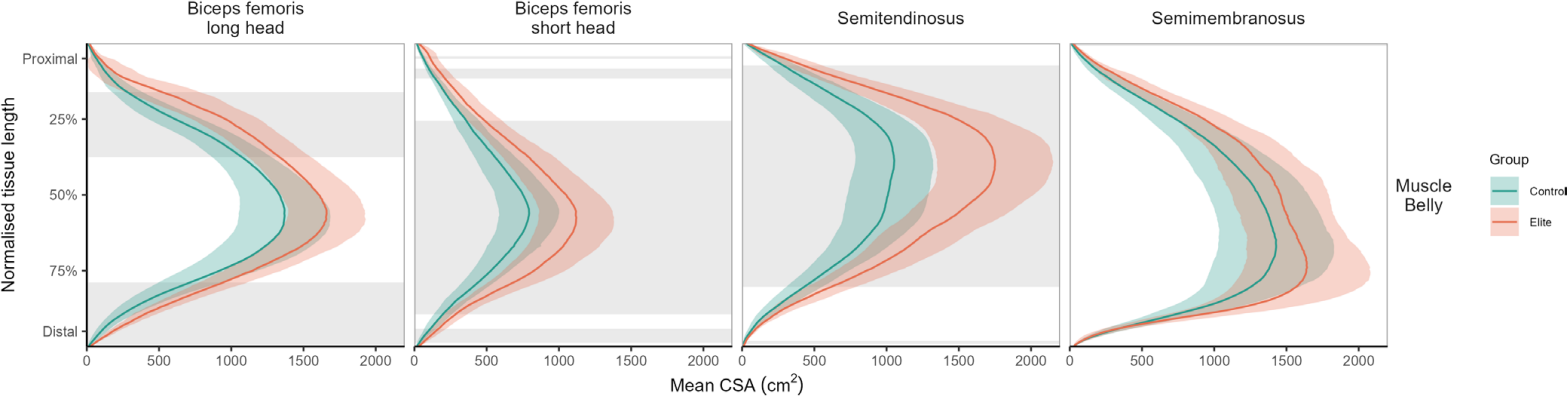
Statistical parametric mapping analysis of muscle cross‐sectional area (CSA) normalized to 0%–100% of its proximal‐to‐distal length. Gray‐shaded areas represent regions of statistical difference between recreationally active control and elite groups.

There was a main effect of group on the mean CSA along the length of the hamstring muscles (Figure 4). In the BFlh, the elite group had significantly larger CSA than the recreationally active group between 16.1%–37.5% (*p* < 0.001) and 78.9%–99.4% (*p* < 0.001) of the proximal‐to‐distal length. The elite group had significantly larger BFsh CSA than the recreationally active group between 4.3%–5.1% (*p* = 0.049), 8.4%–11.7% (*p* = 0.039), 25.6%–89.3% (*p* < 0.001) and 94.2%–98.8% (*p* = 0.030) of the proximal‐to‐distal length. The elite group had significantly larger semitendinosus CSA than the recreationally active group between 7.3%–80.3% (*p* < 0.001) and 98.1%–99.1% (*p* = 0.049) proximal‐to‐distal length. There were no differences along the length for semimembranosus.

### 3.5 Association Between Muscle‐Tendon Unit Characteristics and Performance

For all participants pooled, stepwise forward regression revealed that the variance in maximal sprint velocity was explained by the combination of variance in semitendinosus and semimembranosus muscle and tendon volumes and BFlh carnosine content (Adjusted *R* ^2^ = 0.648) (Table 3). The variance in maximal eccentric knee flexor force was explained by a combination of variance in BFlh and semimembranosus muscle volumes, and semitendinosus tendon volume (Adjusted *R* ^2^ = 0.588). For the elite group only, the variance in maximal sprint velocity was explained by the variance in BFlh muscle and tendon volume, BFsh tendon volume, semimembranosus muscle volume, and semitendinosus tendon volume (Adjusted *R* ^2^ = 0.605). The variance in maximal eccentric knee flexor force was explained by the variance in BFlh muscle volume, and semimembranosus and semitendinosus tendon volumes (Adjusted *R* ^2^ = 0.844). For the control group only, the variance in maximal sprint velocity was explained by the variance in BFsh tendon volume, semimembranosus muscle volume, and semitendinosus muscle and tendon volume (Adjusted *R* ^2^ = 0.797). The variance in maximal eccentric knee flexor strength was explained by variance in BFsh muscle volume (Adjusted *R* ^2^ = 0.332). Fitted regression model equations are presented in Supporting Information S3.

**TABLE 3:** Forward stepwise regression results, showing the variables selected in the regression, the adjusted *R* ^2^ value, degrees of freedom (df), *F* statistic, and *p* value.

	Variables selected	Adjusted *R* ^2^	df	*F*	*p* value
Pooled participants					
Peak eccentric knee flexor force	BFlh musc SM musc ST tend	0.588	3,26	14.815	< 0.001
Maximal sprint velocity	SM musc SM tend ST musc ST tend BFlh carn	0.648	5,19	9.832	< 0.001
Elite					
Peak eccentric knee flexor force	BFlh musc SM tend ST tend	0.844	3,11	26.229	< 0.001
Maximal sprint velocity	BFlh musc BFlh tend BFsh tend SM musc ST tend	0.605	5,7	4.669	0.034
Recreationally active					
Peak eccentric knee flexor force	BFsh musc	0.332	1,13	7.972	0.014
Maximal sprint velocity	BFsh tend SM musc ST musc ST tend	0.797	4,7	11.815	0.003

Abbreviations: BFlh, biceps femoris long head; BFsh, biceps femoris short head; carn, estimated carnosine; musc, muscle volume; SM, semimembranosus; ST, semitendinosus; tend, tendon volume.

## 4 Discussion

### 4.1 Summary of Key Results

The main finding of this study is that the elite sprint and horizontal jump athletes had larger hamstring muscles, aponeuroses, and free tendon volumes than recreationally active individuals, with semitendinosus showing the greatest relative size difference. Regional CSA analysis demonstrated non‐uniform hypertrophy patterns along the length of the hamstring muscles, suggesting region‐specific adaptations. Importantly, despite the elite group possessing a larger BFlh aponeurosis interface area, proportional enlargement of the BFlh aponeuroses and muscle was evident as the BFlh muscle volume‐to‐interface area ratio was not significantly different between groups. Further, the moderate correlation between BFlh aponeurosis interface areas and muscle volume indicates a positive relationship between tissue sizes. This study is the first to apply proton magnetic resonance spectroscopy to estimate BFlh muscle typology, providing a promising non‐invasive method for future work. The elite athletes displayed a greater estimated proportion of Type II fibers, although this was not associated with interface area. When combined, the volumes of medial hamstring muscles and tendons, along with muscle typology, accounted for the greatest variation in maximal sprint velocity for all participants. Additionally, BFlh and semimembranosus muscle volumes alongside semitendinosus tendon volume explained the most variance in eccentric knee flexor strength. These findings highlight the importance of hamstring muscle‐tendon unit geometry and muscle typology in athletic performance.

### 4.2 Differences in Muscle Volumes and Regional CSA

Hamstring muscle volume was non‐uniformly larger in the elite group than the recreationally active group. The largest difference between groups was observed for semitendinosus, which was 38% larger in the elite sprinters/jumpers than recreationally active individuals. Elite athletes also displayed significantly larger BFlh (22%) and BFsh muscles (34%) than recreationally active athletes. Due to the high strain experienced by biceps femoris and the relatively greater lengthening velocities of semitendinosus during running [32, 33], sprint training may lead to preferential hypertrophy of these muscles. These findings are consistent with prior studies [5, 6, 10]. For example, Handsfield et al. observed that the semitendinosus was 54% larger and both BFlh and BFsh 26% larger in sprinters than in non‐sprinters [5]. Differences in the semimembranosus volumes were relatively smaller than for other muscles but similar between studies (Handsfield = 20%, present study = 17%) [5]. Notably, differences in muscle morphology were heterogenous along the length of the hamstrings. Elite athletes typically displayed larger proximal and distal regions of the BFlh than recreationally active athletes, while CSA was uniformly larger along the length of BFsh and ST; a pattern that aligns with findings from Kawama et al. (2021). Although the reasons for these regional differences are unclear, BFsh and semitendinosus have longer fascicles, in comparison to the relatively shorter fascicles of the BFlh [34] which may facilitate regional adaptation. Therefore, the spread of hypertrophy along the length in the current study challenges previous assumptions of uniform hypertrophy and highlights the need for further investigation.

### 4.3 Tendon and Aponeurosis Geometry

This study is the first to show that hamstring free tendons and aponeuroses were 22%–41% larger in elite sprinters and jumpers than recreationally active athletes (Supporting Information S5). The BFlh and semimembranosus proximal aponeuroses—muscles with relatively large hip joint moment arms [35]—were significantly larger in the elite group than recreationally active group. The BFsh distal aponeurosis and semitendinosus distal free tendon were also larger in the elite group, and these muscles have larger moment arms at the knee [35]. Larger muscles with larger tendons may reflect adaptations to the mechanical demands placed on the joints they span. Specifically, increased tendon/aponeurosis size may support greater hip extensor torques (via semimembranosus and BFlh) or knee flexor torques (via semitendinosus and BFsh) during high‐speed running. Similar hypertrophy has been observed in the Achilles and patellar tendons, and vastus lateralis aponeuroses [21], after long‐term training [22, 23], suggesting that habitual loading plays a significant role in tendon size. Notably, the elite group produced significantly greater knee flexor force (Table 2), indicating the potential functional relevance of these larger muscle‐tendon units. Larger aponeuroses provide a greater area for force distribution, potentially decreasing stress within the structure. Furthermore, previous research suggests narrower BFlh aponeuroses may increase muscle fiber strain near the MTJ [18, 19]. However, in this study, the BFlh muscle volume‐to‐interface area ratio did not differ between groups and the moderate correlation between the two tissues suggests proportional enlargement or scaling of aponeurosis interface area in relation to muscle volume. Despite the lack of difference between groups, it is worth noting that variation within groups was still apparent. For example, the elite group possessed a range of aponeurosis areas for similar muscle volumes. The implications of such variation is ultimately unknown, but the findings of the present study demonstrate the importance of considering both muscle and tendon adaption to training, as both are related to athletic performance.

### 4.4 Muscle Typology

Elite athletes displayed a greater estimated proportion of Type II fibers than the recreationally active group which may have implications for performance and injury risk. Lievens et al. [24] reported a 5.4‐fold higher risk of sustaining a HSI in athletes with a greater proportion of fast fibers compared to those with slow typology. This may, in part, be a result of Type II fibers' lower tolerance to fatigue than Type I fibers [36], and physiological differences at the sarcomere level, such as smaller or less elastic forms of structural proteins [37] which may increase susceptibility to damage during eccentric exercise. However, BFlh muscle typology may also have implications for stress and strain experienced at the MTJ. Jakobsen et al. [25] reported differences between fiber types regarding their footprints at the MTJ (i.e., the area of an individual fiber's insertion), and hypothesized that the smaller footprint of Type II fibers might increase stress and strain at the interface. While the present study found no significant association between BFlh carnosine content and interface area, the reported interface area in the present study does not capture the complex interdigitations of the MTJ [38], or the direct footprint of individual fiber types. Future work is needed to determine the interactions between muscle typology and interface area and its effect on injury risk.

### 4.5 Strength and Sprint Performance

Muscle‐tendon geometry and composition was significantly associated with sprint performance and maximal strength. The variance in maximal sprint velocity was mostly (65%) explained by a combination of BFlh muscle typology and medial hamstring muscle and tendon volumes; indeed, semimembranosus muscle and semitendinosus tendon volumes were consistent predictor variables for maximal sprint velocity in all regression models. Type II fibers generate more power at a given force than Type I [39], thereby supporting the rationale that possessing a higher proportion of Type II fibers is advantageous for sprint performance. Semitendinosus has longer, less pennate fibers than the other hamstring muscles [34], which is considered favorable for higher contraction velocities [32]. Further, during the stance phase of high‐speed running, semimembranosus experiences greater forces and performs more mechanical work during swing than the other biarticular hamstrings [33]. This may indicate a greater role for the semimembranosus in forward propulsion during high‐speed running.

The variance in semitendinosus total tendon volume (i.e., distal aponeurosis and free tendon volumes combined), and semimembranosus and BFlh muscle volumes explained 59% of the variance in eccentric knee flexor strength of all participants. Muscle and/or tendon volumes of BFlh, semimembranosus and semitendinosus were implicated as predictor variables for the pooled and elite regression models. Semimembranosus has the greatest physiological CSA of the hamstring muscles, followed by BFlh, with both possessing long tendons which may explain their greater contributions to force production [32, 34]. The Nordic is also known to be medial hamstring dominant, with higher activity in the semitendinosus [40, 41], so it is possible that having larger distal tendon volumes for a knee‐dominant test is favorable.

### 4.6 Limitations and Future Research

This study's cross‐sectional design limits firm conclusions regarding the effect of training history on geometric outcomes. There is currently an absence of long‐term (> 14 weeks), well‐controlled trials which report tendon‐related outcomes [20]. Therefore, future work may focus on determining the time‐course of adaptation over long durations. Further, adaptation of material and mechanical properties of hamstring free tendons and aponeuroses to short‐ and long‐term exposure to mechanical loading are also unknown. Currently, methods to directly establish such changes in the hamstrings remain limited. Tendon mechanical properties adapt faster and plateau earlier than geometry (i.e., CSA) [42]. Consequently, long‐term trained athletes likely possess altered material and mechanical properties alongside larger muscle‐tendon units. The effect of larger muscle and tendon volumes, interface areas and a greater proportion of Type II fibers on stress and strain experienced by the hamstrings requires exploration to understand potential responses to injurious mechanisms and adaptation to exercise. While muscle volume has been used in ratio calculations in the current study, volume is influenced by muscle length which may not directly contribute to strain experienced by the proximal aponeurosis. To determine a more accurate ratio between muscle force generating capacity and aponeurosis size, future work should incorporate measures of physiological CSA or equivalent. The direct measurement of muscle typology derived from a muscle biopsy may have provided further insight into the relationships between pure and hybrid fibers and the performance and muscle‐tendon geometry measures, but its invasive nature can be prohibitive, particularly in elite populations. While there is some evidence to indicate that carnosine‐derived measures provide a strong estimation of muscle typology in the gastrocnemius [43] and vastus lateralis [44], these comparisons have not been established in the BFlh. Future work is also needed to determine if training interventions that alter both hamstring muscle and tendon lead to improvements in performance. While the recreationally active cohort was older than the elite athletes, there is little reason to suspect these differences in biological age—rather than training age/history—meaningfully affected the results of the study. However, future studies may wish to match participant characteristics more closely to eliminate any possible confounding effects. This study also contained relatively few female participants and larger, sex‐balanced samples may be better suited to exploring interactions between physical characteristics and performance. The addition of a familiarization session may have been beneficial to ensure participants had a consistent level of experience with sprint and strength testing protocols prior to data collection. However, elite participants were already familiar and the recreationally active group received standardized coaching during warm‐up trials and strong encouragement during experimental testing.

## 5 Conclusion

Elite level sprint and horizontal jump athletes are faster, stronger and possess larger hamstring muscle and tendon‐aponeurosis structures, larger BFlh interface areas, and a higher estimated proportion of BFlh Type II fibers than recreationally active individuals. Variance in medial hamstring muscle and tendon volumes combined with BFlh typology explained the greatest variance in sprint velocity, while BFlh and semimembranosus muscle volumes and semitendinosus tendon volume explained the greatest variance in eccentric knee flexor strength. These findings may have important implications for strategies targeted at enhancing sprint performance and minimizing hamstring injury risk.

## 6 Perspective

Previous research has established that athletic populations display larger hamstring muscles [5, 6, 10]; however, the geometry of the associated tendons has not been clearly characterized until now. In the present study, elite sprinters/jumpers possessed larger hamstrings and were both stronger and faster than recreationally active individuals. Their correspondingly larger aponeuroses and free tendons likely reflect adaptations to withstand the forces associated with high‐level performance. Estimated muscle typology has long been associated with athletic performance, whereby sprint athletes typically possess a greater proportion of Type II ‘fast’ fibers [8]. Tendon properties also influence athletic performance, as stiffer tendons are associated with greater rates of force development [15]. The current findings add to this understanding by demonstrating that hamstring tendon volumes are associated with both sprint velocity and peak eccentric knee flexor strength, while estimated muscle typology is associated with sprint velocity alone. These results suggest that hamstring tendon geometry, in addition to muscle morphology and composition, plays an important role in supporting athletic performance.

## Ethics Statement

Ethical approval was granted by the Griffith University Human Research Ethics Committee (reference number: GU 2020/508).

## Consent

Written informed consent was provided by all participants prior to data collection.

## Conflicts of Interest

A/Prof Bourne is supported by an Advance Queensland Mid‐Career Industry Research Fellowship in partnership with VALD. Prof David Opar is listed as a co‐inventor on a patent (PCT/AU2012/001041.2012), filed by the Queensland University of Technology (QUT), for a field‐testing device of eccentric hamstring strength, which is now known commercially as the NordBord. Prof Opar has received revenue distributions from QUT based on revenue that QUT has generated through the commercialisation of his intellectual property. Prof Opar is a minority shareholder in Vald Performance Pty Ltd., the company responsible for commercialization of the NordBord, among other devices. Prof Opar has received research funding from Vald Performance for work unrelated to the current manuscript. Prof Opar was previously the Chair of the Vald Performance Research Committee, a role that was unpaid. Prof Opar has family members who are minor shareholders and/or employees of Vald Performance. The remaining authors have no conflicts of interest to declare.

## Supporting information


**Appendix S1:** sms70151‐sup‐0001‐AppendixS1.docx.

## Data Availability

The data that support the findings of this study are available from the corresponding author upon reasonable request.
