# Supplementary material for: Differences in Hamstring Muscle‐Tendon Unit Geometry and Function Between Elite Sprint and Jump Athletes and Recreationally Active Controls
Source: Scand J Med Sci Sports. 2025 Oct 24;35(11):e70151. doi: 10.1111/sms.70151 (PMC12552802; doi:10.1111/sms.70151)
Supplement: Supplementary file 1 — Appendix S1: sms70151‐sup‐0001‐AppendixS1.docx. [file SMS-35-e70151-s001.docx]

TITLE

Differences in hamstring muscle-tendon unit geometry and function between elite sprint and jump athletes and recreationally active controls

JOURNAL

Scandinavian Journal of Medicine and Science in Sports

**AUTHORS**

Lazarczuk, Stephanie L. ^1,2,3^ (ORCID: 0000-0001-8467-8799)

Hams, Andrea H. ^2,3^ (ORCID: 0000-0003-2908-4271)

Bellinger, Phillip M. ^2,4^ (ORCID: 0000-0001-7266-2976)

Timmins, Ryan G. ^5,6^ (ORCID**: 0000-0003-4964-1848)**

**Lievens, Eline ^7^ (ORCID:** 0000-0003-2549-3369**)**

**Kennedy, Ben ^8^ (ORCID: 0009-0002-1410-9843)**

Opar, David ^6,9^ (ORCID: 0000-0002-8354-6353)

Barrett, Rod S. ^2,3^ (ORCID: 0000-0002-1784-1629)

Bourne, Matthew N. ^2,3^ (ORCID: 0000-0002-3374-4669)

AFFILIATIONS

1. Department of Sport and Health, Southampton Solent University, Southampton, United Kingdom
2. School of Health Sciences and Social Work, Griffith University, Gold Coast, QLD, Australia
3. Australian Centre for Precision Health and Technology, Griffith University, Gold Coast, QLD, Australia
4. Griffith Sports Science, Griffith University, Gold Coast, QLD, Australia
5. School of Behavioural and Health Sciences, Australian Catholic University, Brisbane, QLD, Australia
6. Sports Performance, Recovery, Injury and New Technologies (SPRINT) Research Centre, Australian Catholic University, Melbourne, VIC, Australia
7. Department of Movement and Sport Sciences, Ghent University, Ghent, Belgium
8. Mermaid Beach Radiology, Gold Coast, Australia
9. School of Behavioural and Health Sciences, Australian Catholic University, Melbourne, VIC, Australia

CORRESPONDING AUTHOR

Stephanie L. Lazarczuk

Department of Sport & Health,

Southampton Solent University,

East Park Terrace,

Southampton,

SO14 0YN

United Kingdom

[Stephanie.lazarczuk@solent.ac.uk](mailto:Stephanie.lazarczuk@solent.ac.uk)

**S1. Extended participant characteristics.**

| **No.** | **M/F** | **Age** | **Height** | **Mass** | **Events** | **PB** | **Training per week** |
| --- | --- | --- | --- | --- | --- | --- | --- |
| **ELITE** | | | | | | | |
| 1 | M | 25 | 187 | 85 | Triple Jump | 16.28 m | 2 x jumps, 1 x running, 1 x gym |
| 2 | M | 20 | 184 | 65 | 400 m | 49.11 s | 3 x track, 3 x gym |
| 3 | M | 22 | 179 | 66 | 400 m  800 m | 50.51 s  1:56:32 min | 6 x p/wk |
| 4 | M | 21 | 184 | 78 | Long jump | 7.97 m | 2 x hills, 2 x gym, 1 x jump |
| 5 | M | 24 | 182 | 73 | 400 m | 47.0 s | 6 x p/wk |
| 6 | M | 22 | 177 | 76 | 100 m  200 m | 10.68 s  21.68 s | 4 x track, 2-3 x gym |
| 7 | F | 19 | 173 | 60 | 400 m | 55.07 s | 7 x p/wk |
| 8 | M | 24 | 180 | 69 | Long jump | 7.74 m | 6 x p/wk |
| 9 | M | 18 | 180 | 73 | 110 m hurdles  400 m hurdles | 15.4 s  57.3 s | 6 x p/wk |
| 10 | M | 25 | 192 | 88 | Long jump | 7.90 m | 2 x strength/gym, 4 x jump/sprints |
| 11 | M | 23 | 188 | 78 | 400 m | 48.71 | 6 x p/wk |
| 12 | M | 20 | 190 | 76 | Long jump | 7.84 m | 2 x gym, 2 x plyo, 2 x jumps |
| 13 | M | 21 | 185 | 81 | Long jump  100 m | 7.83 m  10.52 s | 3 x gym, 4 x track |
| 14 | F | 22 | 160 | 50 | 100 m | 12.45 s | 4 x track, 2 x gym |
| 15 | F | 19 | 178 | 55 | Long jump | 6.49 m | 3 x gym, 2 x sprint, 2 x jumps |
| **RECREATIONALLY ACTIVE** | | | | | | | |
| 16 | M | 21 | 185 | 78.8 | - | - | Sporadic |
| 17 | M | 26 | 176 | 71 | - | - | 3 x surf |
| 18 | M | 26 | 175 | 78 | - | - | 3 x 5km run |
| 19 | M | 28 | 175 | 66 | - | - | 2 x 60 min HIIT |
| 20 | M | 25 | 190.5 | 86 | - | - | 1 x exercise class |
| 21 | M | 28 | 188 | 126 | - | - | 2 x 40 min weights class, plus work manual labour |
| 22 | M | 24 | 187 | 87 | - | - | Work manual labour |
| 23 | M | 21 | 180 | 70 | - | - | 1 x volleyball social |
| 24 | F | 27 | 165 | 61 | - | - | Sporadic cardio or gym |
| 25 | M | 25 | 176 | 91.5 | - | - | <1 x circuit class |
| 26 | F | 32 | 158 | 60 | - | - | 3 x generic gym |
| 27 | M | 28 | 177 | 72 | - | - | 1 x gym, 1 x 30km bike, 1 x 5km run |
| 28 | F | 23 | 171 | 65 | - | - | Sporadic |
| 29 | M | 23 | 176 | 80 | - | - | Sporadic |
| 30 | F | 28 | 161 | 52 | - | - | 3 x generic gym |

Note: F = female; M = male; PB = personal best; p/wk = per week

**S2. Reliability and coefficient of variation of magnetic resonance spectroscopy in the BFlh**

Given that this is the first study to apply magnetic resonance spectroscopy to the BFlh, reliability testing was conducted. BFlh carnosine content was assessed twice in 7 additional healthy, and recreationally active, control individuals (3 female, 30.9 ± 4.9 years, 171.6 ± 12.6 cm, 73.6 ± 14.9 kg). Participants attended a single testing session and were required to stand between two acquisition sequences before being repositioned in the scanner.

A two-way, mixed effects, absolute agreement intraclass correlation coefficient (ICC_3,1_) was calculated for the carnosine peak-to-water peak value. Moderate reliability was found for the carnosine peak-to-water peak value (ICC_3,1_ = 0.576, *p* = 0.094). The average coefficient of variation (CV) between repeat measures of the carnosine peak-to-water peak value was 11%.

**S3a. Fitted regression model equations for maximal sprint velocity and maximal knee flexor force for the pooled participants.**

$$\boldsymbol{Maximal sprint velocity} =2.854+\left( 0.0013 \times semitendinosus muscle volume \right)+\left( 0.329 \times semitendinosus tendon volume \right)+\left( 0.0047 \times BFlh carnosine peak to water peak \right)+\left( 0.0062 \times semimembranosus muscle volume \right)+(0.093 \times semimembranosus tendon volume)$$

$$\boldsymbol{Maximal eccentric knee flexor force} = -119.29+\left( 0.741 \times BFlh muscle volume \right)+\left( 33.12 \times semitendinosus tendon volume \right)+(0.527 \times semimembranosus muscle volume)$$

**S3b**. **Fitted regression model equations for maximal sprint velocity and maximal knee flexor force for the elite participants.**

$$\boldsymbol{Maximal sprint velocity} =7.904+\left( 0.015 \times semimembranosus muscle volume \right)+\left( -0.421 \times BFsh tendon volume \right)+\left( 0.119 \times BFlh tendon volume \right)+\left( 0.291 \times semitendinosus tendon volume \right)+(-0.010 \times BFlh muscle volume)$$

$$\boldsymbol{Maximal eccentric knee flexor force} = -367.55+\left( 2.262 \times BFlh muscle volume \right)+\left( 91.549 \times semitendinosus tendon volume \right)+(-17.375 \times semimembranosus tendon volume)$$

**S3c**. **Fitted regression model equations for maximal sprint velocity and maximal knee flexor force for the control participants.**

$$\boldsymbol{Maximal sprint velocity} =4.230+\left( 0.105 \times semitendinosus tendon volume \right)+\left( 0.018 \times semimembranosus muscle volume \right)+\left( -0.012 \times semitendinosus muscle volume \right)+\left( 0.399 \times BFsh tendon volume \right)$$

$$\boldsymbol{Maximal eccentric knee flexor force} = 59.672+\left( 1.912 \times BFsh muscle volume \right)$$

**S4.** **The relationship between gastrocnemius carnosine concentration and BFlh carnosine peak-to-water peak**

The relationship between the gastrocnemius carnosine concentration and the BFlh carnosine peak-to-water peak for the elite and recreationally active groups was determined using a Spearman-rank correlation coefficient. In the elite group, a significant, strong, positive correlation between the BFlh carnosine peak-to-water peak value and the gastrocnemius carnosine concentration was found (rho = 0.745, *p* = 0.001; Figure D2). In the recreationally active group, a significant, moderate, positive correlation between the BFlh carnosine peak-to-water peak value and the gastrocnemius carnosine concentration (rho = 0.662, *p* = 0.012, Figure D2). The strong correlation between gastrocnemius carnosine concentration and the BFlh carnosine peak-to-water peak suggests that the application of proton magnetic resonance spectroscopy in the BFlh construct validity, since the gastrocnemius carnosine content has previously been interrogated as reliable and valid (Baguet et al., 2011; Bellinger et al., 2020) .


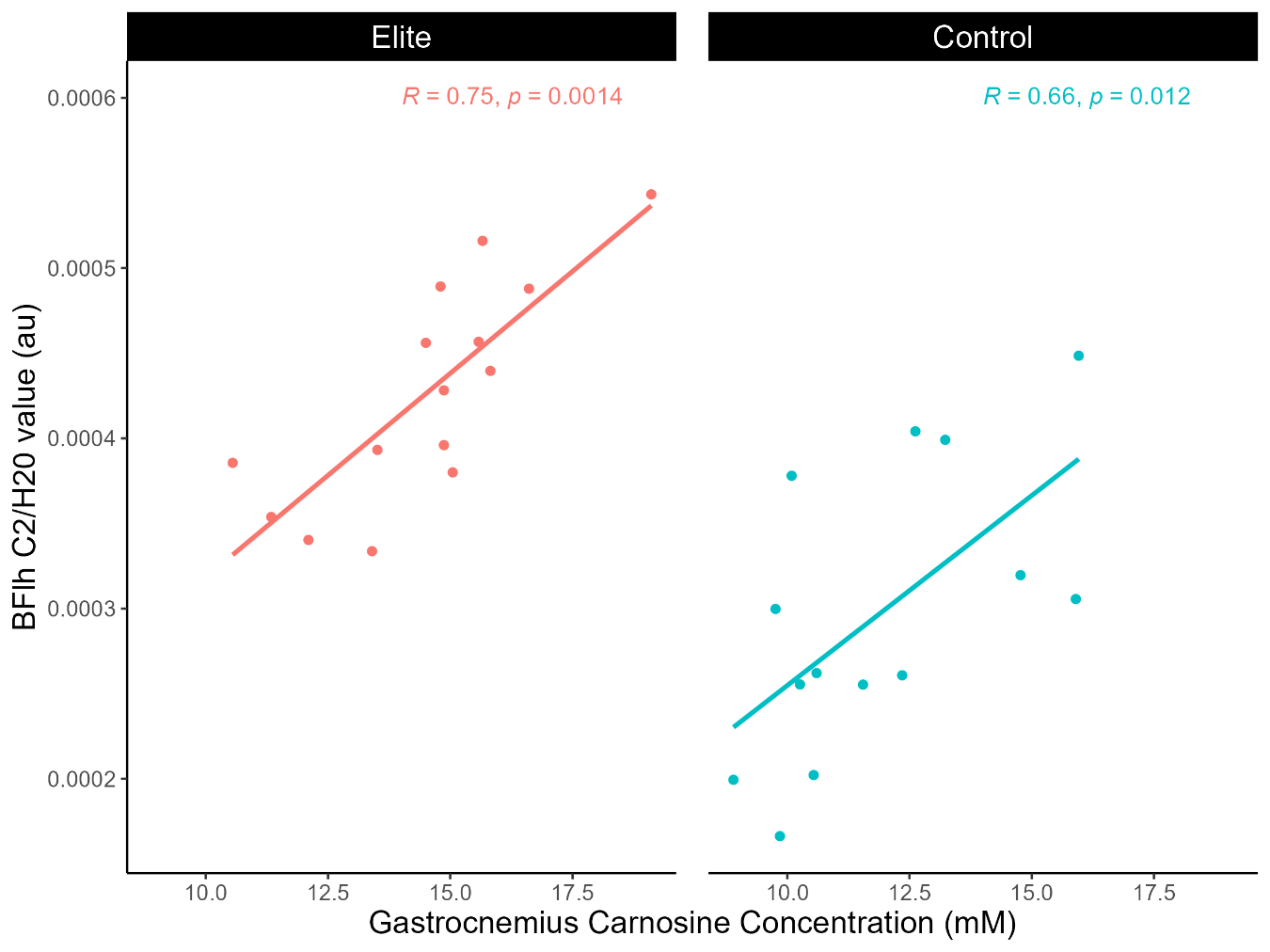


**Figure S4.1**. Relationship between gastrocnemius carnosine concentration and biceps femoris long head (BFlh) carnosine peak-to-water peak value (C2/H20) in the recreationally active control and elite groups, derived from proton magnetic resonance spectroscopy.

**Table S5.1**. **Absolute volumes (cm^3^; mean ± standard deviation) by tissue, location, and group showing the percent by which the elite group is larger than the recreationally active group.**

| Muscle | Location | Elite | Recreationally active | % |
| --- | --- | --- | --- | --- |
| Biceps femoris long head | Proximal free tendon | 1.03 ± 0.46 | 0.74 ± 0.39 | 28 |
|  | Proximal aponeurosis | 3.22 ± 0.97 | 2.44 ± 0.62 | 24 |
|  | Muscle | 282.80 ± 46.93 | 221.50 ± 55.58 | 22 |
|  | Distal aponeurosis | 6.28 ± 2.40 | 3.76 ± 1.80 | 40 |
|  |  |  |  |  |
| Biceps femoris short head | Muscle | 171.28 ± 44.69 | 113.67 ± 33.30 | 34 |
|  | Distal aponeurosis | 4.23 ± 1.32 | 2.77 ± 0.82 | 35 |
|  | Distal free tendon | 0.27 ± 0.16 | 0.19 ± 0.12 | 30 |
|  |  |  |  |  |
| Semimembranosus | Proximal free tendon | 2.55 ± 0.94 | 1.79 ± 0.68 | 30 |
|  | Proximal aponeurosis | 5.62 ± 1.50 | 3.30 ± 1.15 | 41 |
|  | Muscle | 318.83 ± 69.90 | 265.53 ± 66.15 | 17 |
|  | Distal aponeurosis | 6.31 ± 1.69 | 4.93 ± 1.19 | 22 |
|  | Distal free tendon | 0.48 ± 0.22 | 0.27 ± 0.16 | 29 |
|  |  |  |  |  |
| Semitendinosus | Muscle | 349.04 ± 85.41 | 217.38 ± 57.59 | 38 |
|  | Distal aponeurosis | 2.64 ± 0.67 | 1.85 ± 0.73 | 30 |
|  | Distal free tendon | 1.49 ± 0.43 | 0.94 ± 0.40 | 37 |

**Table S5.2**. **Average cross-sectional area (cm^2^; mean ± standard deviation) by tissue, location, and group.**

| Muscle | Location | Elite | Recreationally active |
| --- | --- | --- | --- |
| Biceps femoris long head | Proximal free tendon | 0.16 ± 0.06 | 0.14 ± 0.06 |
|  | Proximal aponeurosis | 0.15 ± 0.06 | 0.11 ± 0.05 |
|  | Muscle | 9.37 ± 5.84 | 7.22 ± 5.27 |
|  | Distal aponeurosis | 0.35 ± 0.15 | 0.21 ± 0.13 |
|  |  |  |  |
| Biceps femoris short head | Muscle | 6.15 ± 3.91 | 4.22 ± 2.85 |
|  | Distal aponeurosis | 0.20 ± 0.12 | 0.14 ± 0.07 |
|  | Distal free tendon | 0.12 ± 0.05 | 0.09 ± 0.05 |
|  |  |  |  |
| Semimembranosus | Proximal free tendon | 0.22 ± 0.10 | 0.18 ± 0.07 |
|  | Proximal aponeurosis | 0.25 ± 0.10 | 0.16 ± 0.07 |
|  | Muscle | 10.37 ± 6.02 | 8.85 ± 5.45 |
|  | Distal aponeurosis | 0.34 ± 0.12 | 0.25 ± 0.11 |
|  | Distal free tendon | 0.12 ± 0.05 | 0.08 ± 0.04 |
|  |  |  |  |
| Semitendinosus | Muscle | 10.20 ± 6.41 | 6.44 ± 4.05 |
|  | Distal aponeurosis | 0.19 ± 0.08 | 0.13 ± 0.07 |
|  | Distal free tendon | 0.10 ± 0.03 | 0.08 ± 0.04 |
